# Reoperation Strategy for Failure of Cervical Disc Arthroplasty at Index and Adjacent Levels

**DOI:** 10.3390/jcm14062038

**Published:** 2025-03-17

**Authors:** Chae-Gwan Kong, Jong-Beom Park

**Affiliations:** Department of Orthopaedic Surgery, Uijeongbu St. Mary’s Hospital, The Catholic University of Korea College of Medicine, Uijeongbu 11765, Republic of Korea; gongjae@catholic.ac.kr

**Keywords:** reoperation strategy, failure, cervical disc arthroplasty, index and adjacent levels

## Abstract

Cervical disc arthroplasty (CDA) is a motion-preserving alternative to anterior cervical discectomy and fusion (ACDF) for cervical degenerative disease, reducing adjacent segment degenerative disease (ASD). Despite its benefits, some patients experience CDA failure due to prosthesis-related complications, heterotopic ossification, segmental kyphosis, ASD, or facet joint degeneration, necessitating revision surgery. Reoperation strategies depend on the failure mechanism, instability, sagittal malalignment, and neural compression. Anterior revision is suited for prosthesis failure, recurrent disc herniation, or ASD, enabling prosthesis removal, decompression, and fusion. In select cases, reimplantation may restore motion. Posterior approaches are preferred for facet degeneration, multilevel stenosis, or posterior hypertrophy, with options including foraminotomy, laminoplasty, or laminectomy and fusion. Complex cases may require combined anterior and posterior surgery for optimal decompression and stability. This narrative review outlines revision strategies, emphasizing biomechanical assessment, radiographic evaluation, and patient-specific considerations. Despite surgical challenges, meticulous planning and execution can optimize outcomes.

## 1. Introduction

Cervical disc arthroplasty (CDA) has been widely adopted as a motion-preserving alternative to anterior cervical discectomy and fusion (ACDF) for treating cervical degenerative disc disease [1,2,3,4]. While CDA has demonstrated favorable outcomes in appropriately selected patients [5,6,7,8,9,10,11,12], long-term follow-up studies indicate that some patients experience complications necessitating revision surgery. While many of these complications can be managed conservatively, a subset of patients experiences persistent symptoms or progressive deterioration that necessitate revision surgery [13,14,15,16,17,18,19,20,21]. However, a significant lack of consensus remains on the optimal strategies for managing CDA failure.

Previous reviews on CDA failure and revision surgery have focused on case series, retrospective studies, and expert opinions without systematically comparing different revision strategies in a structured format. Additionally, prior literature has often addressed CDA failure as a secondary topic within broader discussions on motion-preserving cervical spine surgery rather than conducting a dedicated, in-depth analysis. Existing studies focus on isolated aspects of CDA revision, such as heterotopic ossification, adjacent segment disease, or implant failure, without comprehensively evaluating all available revision techniques [22,23,24,25,26].

Several factors contribute to the challenges in revising failed CDA, including implant migration, subsidence, heterotopic ossification, and progressive adjacent segment disease. Unlike primary CDA procedures, revision surgeries often necessitate additional stabilization strategies through anterior revision, posterior fusion, or a combined approach. However, the literature on revision CDA remains fragmented, relying heavily on retrospective case series and expert opinions rather than high-quality comparative studies or randomized controlled trials [27,28,29].

Given the heterogeneity of studies related to CDA failure, a systematic review with meta-analysis was not feasible due to the following reasons: (1) Lack of high-level evidence: most published studies on CDA failure consist of case series, retrospective cohort studies, and expert consensus reports rather than randomized controlled trials. (2) Variability in failure mechanisms and revision techniques: CDA failures arise from multiple causes, including prosthesis-related complications, biological responses, and progressive degeneration, making it difficult to apply a uniform analysis. (3) Diversity in patient selection and surgical decision-making: surgeons employ different revision strategies based on implant type, patient anatomy, and the severity of the failure, leading to inconsistencies in outcome reporting.

This narrative review aims to address these challenges: (1) Synthesize current evidence on CDA failure mechanisms and revision techniques. (2) Identify gaps in high-quality data and highlight areas requiring further research. (3) Provide practical guidance for surgeons by summarizing indications, contraindications, and surgical considerations for revision CDA. (4) Compare different revision strategies (anterior, posterior, and combined approaches) and their long-term clinical outcomes. By consolidating the available literature, this review serves as a comprehensive reference for spine surgeons encountering CDA failure and requiring structured decision-making frameworks.

## 2. Methods

### 2.1. Literature Selection Process

This study is a narrative review of the current literature on revision strategies for failed cervical disc arthroplasty (CDA). A structured literature search was conducted across multiple databases to ensure a comprehensive synthesis of available evidence. Given the heterogeneity of studies on CDA failure and revision, a systematic review with meta-analysis was not feasible, as most available studies consist of retrospective case series, expert opinions, and small cohort studies rather than high-quality randomized controlled trials.

### 2.2. Database Search Strategy

We conducted a comprehensive literature search using the following electronic databases: PubMed, Scopus, and Web of Science electronic databases.

### 2.3. Search Terms and Keywords

The literature search was performed using the following keywords and Boolean operators:

1. “Cervical disc arthroplasty failure” OR “CDA revision surgery”.
2. “Adjacent segment disease after CDA”.
3. “Reoperation strategies for failed cervical disc replacement”.
4. “Biomechanical considerations in CDA revision”.
5. “Anterior vs. posterior approach for CDA failure”.
6. “Complications of cervical disc arthroplasty”.

### 2.4. Inclusion Criteria

Studies were included if they met the following criteria:

1. Published in peer-reviewed journals (conference abstracts and non-peer-reviewed literature were excluded).
2. Focused on cervical disc arthroplasty failure and revision strategies, including both anterior and posterior surgical approaches.
3. Included surgical outcome data, whether through retrospective case series, prospective cohort studies, or comparative studies.
4. Presented long-term follow-up results related to CDA reoperation and patient-reported outcomes.
5. Written in English (due to feasibility constraints regarding translation and interpretation).

### 2.5. Exclusion Criteria

The following studies were excluded from the review:

1. Studies not explicitly focused on CDA failure or revision (e.g., general cervical spine reviews or primary CDA outcome studies without reoperation data).
2. Articles related only to primary CDA without discussion of revision techniques.
3. Non-English articles without available translations.
4. Case reports with fewer than five patients, as their findings have limited generalizability.
5. Animal or cadaveric studies without direct clinical application.

### 2.6. Study Selection and Screening Process

1. Initial screening was conducted by reviewing titles and abstracts of all retrieved articles.
2. The full text of potentially relevant articles was independently reviewed by two authors to assess eligibility.
3. Discrepancies regarding study inclusion were resolved through discussion, moreover, if necessary, a third senior reviewer was consulted.
4. Additional relevant studies were identified through a manual review of reference lists from selected articles.

## 3. Preoperative Planning for Reoperation of CDA Failure

Reoperation following CDA failure is a complex decision-making process that requires comprehensive preoperative planning. Thorough preparation is critical to the success of any reoperation after CDA failure. A detailed assessment—including clinical evaluation, dynamic radiographic imaging, and biomechanical analysis—is essential to identify the cause of CDA failure and guide the revision strategy [22,23,24,25,26,27,28,29]. Factors such as spinal alignment, instability, bone quality, and patient comorbidities must be carefully considered to minimize the risk of complications and optimize outcomes. Choosing between anterior, posterior, and combined reoperation approaches hinges on meticulously evaluating the patient’s clinical presentation, imaging findings, and biomechanical considerations. A structured approach to preoperative planning ensures optimal surgical results, reduces complications, and effectively addresses the specific cause of CDA failure. The following is a detailed breakdown of the preoperative planning process for reoperation after CDA failure.

### 3.1. Identifying the Cause of CDA Failure

Understanding the underlying cause of CDA failure is the cornerstone of selecting the appropriate surgical approach [13,14,15,16,17,18,19,20,21]. Prosthesis-related complications are the most common causes of CDA failure. Subsidence is the sinking of the prosthesis into the vertebral endplates, leading to instability and neural compression. Migration of dislocation of the prosthesis, either anteriorly or posteriorly, may result in mechanical instability or nerve root impingement. Heterotopic ossification (HO) around the prosthesis can limit motion and increase stress on adjacent segments. Mechanical failure of the artificial disc’s component can lead to prosthesis malfunction.

Regarding biological and degenerative changes contributing to CDA failure, facet joint arthropathy can accelerate degeneration of the facet joints, resulting in axial neck pain or radiculopathy. ASD shows degenerative changes at levels adjacent to the CDA. It can cause radiculopathy or myelopathy [13,14,15,16,17,18]. Ongoing disc degeneration may occur at the index and adjacent levels. Segmental kyphosis due to prosthesis failure or pre-existing alignment issues can lead to intractable axial neck pain and/or neurological symptoms. Spinal instability, such as micromotion or hypermobility at the operated segment, can result in mechanical neck pain. Finally, inadequate decompression during the initial surgery or new compression due to degenerative changes can cause persistent or recurrent radiculopathy and/or myelopathy, leading to CDCA failure requiring revision surgery.

### 3.2. Clinical Evaluation

A detailed clinical assessment helps correlate symptoms with imaging findings and guides the surgical approach [22,23,24,25,26]. Careful history taking is the first step. The onset, duration, and progression of symptoms (neck pain, radiculopathy, and myelopathy) should be evaluated. Prior surgeries, including detailed CDA procedures, should be checked. Previous conservative treatments and their effectiveness are also checked. Any history of trauma or new injuries post-CDA is also important. For physical examination, neurological assessment needs to assess motor strength, sensory deficits, reflex changes, and signs of myelopathy (e.g., hyperreflexia, clonus, gait disturbances). Axial neck pain and radiating pain are assessed and scored with a numeric rating scale. Range of motion is assessed for limitations or pain during cervical movements.

### 3.3. Imaging Studies

High-quality imaging is essential for determining the cause of CDA failure and planning the reoperation strategy. Dynamic (Flexion–Extension) X-rays can assess instability, prosthesis motion, and subsidence. Instability is defined as excessive motion at the operated segment, while subsidence is identified as a loss of disc height compared to postoperative images. A computed tomography (CT) scan helps evaluate bony structures, prosthesis positioning, heterotopic ossification (HO), and facet joint degeneration. HO is graded using the McAfee or Mehren classification systems. Prosthesis malposition is assessed based on the device’s anterior or posterior migration of the device. Facet joint arthropathy is evaluated on the presence of osteophyte formation and joint space narrowing. Magnetic resonance imaging (MRI) can assess neural compression, disc degeneration, and soft tissue pathology, such as ligamentous hypertrophy. In cases where MRI results are inconclusive—especially in the presence of metallic artifacts from the prosthesis—CT myelography can be considered an alternative imaging modality.

### 3.4. Biomechanical and Alignment Considerations

Evaluating the spine’s biomechanical environment is crucial for determining the need for stabilization procedures. Spinal alignment is crucial in determining the success of cervical disc arthroplasty (CDA) and its subsequent revision. Malalignment is a common contributing factor to CDA failure, leading to progressive kyphosis, adjacent segment disease, and mechanical overload on prosthetic devices. Sagittal balance considerations are essential in guiding revision strategies, as improper alignment can lead to postoperative complications, suboptimal fusion, and recurrence of symptoms.

#### 3.4.1. Key Sagittal Balance Parameters

When evaluating patients for CDA revision, several spinal alignment parameters should be considered:

(a) The Sagittal Vertical Axis (SVA) is a key indicator of overall spinal balance. A high SVA (greater than 4 cm) is associated with increased stress on adjacent segments and higher revision rates. Correcting excessive positive SVA often requires posterior fusion to restore sagittal balance.
(b) C2–C7 Lordosis: The usual range of cervical lordosis varies but is typically −20 to −40 degrees. Losing lordosis contributes to segmental kyphosis, leading to anterior shifting of the head and excessive strain on the cervical musculature. Patients with significant loss of lordosis may require posterior instrumentation to restore normal curvature.
(c) T1 Slope: T1 slope correlates with cervical alignment and compensatory changes in the upper thoracic spine. A high T1 slope (>30 degrees) is often associated with increased cervical lordosis requirements. A mismatch between T1 slope and cervical lordosis is a risk factor for adjacent segment disease. Anterior revision is preferred in local subsidence or mild malalignment cases, while posterior fusion is more effective for cases with severe T1 slope abnormalities.

#### 3.4.2. Literature Support and Reference to Kato et al. [30]

Our expanded discussion on spinal alignment considerations in CDA revision is supported by Kato et al., who analyzed biomechanical factors influencing surgical decision-making in degenerative cervical myelopathy. Their findings emphasize the following:

(a) Spinal alignment parameters should guide the selection of revision techniques.
(b) Failure to address sagittal imbalance increases the likelihood of reoperation.
(c) Combining anterior and posterior approaches improves long-term alignment correction in severe cases.

Understanding spinal alignment parameters is essential when planning CDA revision surgery. Sagittal imbalance, particularly positive SVA, decreased lordosis, or high T1 slope, increases failure risks and influences surgical decision-making. Based on preoperative alignment and failure pathology, a personalized surgical approach—whether anterior, posterior, or combined—should be selected based on preoperative alignment and failure pathology.

### 3.5. Risk Factor Assessment

Identifying patient-specific risk factors, such as bone quality, comorbidities, and previous surgeries, helps anticipate complications and tailor the surgical plan [19,20,21,22,23,24,25,26,27]. Osteoporosis or osteopenia increases the risk of prosthesis subsidence and fusion failure. Therefore, bone density testing may be necessary for older patients and those with risk factors for poor bone health. Comorbidities such as smoking, diabetes, and autoimmune disorders can increase the risk of poor wound healing and pseudarthrosis. Scar tissue from prior anterior or posterior procedures can complicate reoperation, thus influencing the choice of surgical approach. Additionally, the possibility of recurrent laryngeal nerve injury from the primary CDA surgery should be evaluated preoperatively through an ENT consultation.

## 4. Determining the Reoperation Strategy for CDA Failure

Reoperation following CDA failure presents unique challenges, differentiating it from revision surgeries in traditional fusion cases [22,23,24,25,26]. The presence of a prosthetic device can alter the biomechanics of the cervical spine and introduce additional considerations regarding implant removal, spinal stability, and deformity correction. Based on the aforementioned evaluations, the most suitable reoperation approach can be selected accordingly. The choice of surgical approach—whether anterior, posterior, or combined—should be tailored to the specific etiology of failure, biomechanical considerations, the patient’s clinical presentation, and radiographic findings. Insights from experts highlight the necessity of a meticulous and individualized approach to optimize surgical outcomes and minimize complications. With careful planning and execution, reoperation after CDA failure can provide significant symptom relief and restore spinal function.

### 4.1. Anterior Reoperation

Anterior reoperation is often preferred for prosthesis-related complications such as subsidence, migration, and mechanical failure [22,23,24,25,26]. It allows for direct removal of the CDA prosthesis, thorough decompression of neural elements, and fusion of the affected segment. Additionally, anterior reoperation is effective in managing ASD and persistent or recurrent disc herniation at the index level. However, anterior reoperation increases the risk of postoperative complications in cases with significant anterior scar tissue from previous surgeries. It cannot address posterior element pathology (e.g., facet arthropathy).

### 4.2. Posterior Reoperation

Posterior reoperation is particularly suited for addressing posterior element pathologies, including facet joint arthropathy, posterior element hypertrophy (ligamentum flavum), or ossification (OPLL), which can cause axial neck pain, radiculopathy, or myelopathy [22,23,24,25,26]. Additionally, multilevel (≥three levels) stenosis extending beyond the index level and kyphotic deformity requiring posterior correction and stabilization is indicated for posterior reoperation. Posterior reoperation techniques, such as laminoplasty, laminectomy, and fusion, provide effective decompression and stabilization, especially in cases where anterior reoperation is contraindicated or has previously failed. Advantages of posterior reoperation include direct decompression of posterior pathologies, the ability to address multilevel disease through laminoplasty or laminectomy, and enhanced stability in cases of segmental kyphosis with posterior fusion. However, posterior reoperation requires more extensive muscle dissection, leading to increased postoperative pain. It can also cause postoperative kyphosis if decompression is performed without fusion.

### 4.3. Combined Anterior–Posterior Reoperation

The cervical spine operates under a delicate balance of stability and mobility, and CDA aims to preserve motion at the affected segment. However, failure of CDA—whether due to implant subsidence, migration, segmental kyphosis, or heterotopic ossification—can disrupt the normal biomechanics of the cervical spine. Revisions following cervical disc arthroplasty (CDA) failure often require complex decision-making, particularly in cases where anterior-only revision may be insufficient to restore stability and correct deformity. This approach allows for adequate decompression and robust stabilization while addressing the unique challenges of CDA failure. Combined anterior–posterior reoperation is indicated in specific cases of CDA failure, including [22,23,24,25,26]:

1. Severe instability or progressive segmental kyphosis is uncorrectable via an anterior-only approach.
2. Implant subsidence or migration causes significant vertebral body compromise.
3. Failed anterior revision surgery, necessitating additional posterior support.
4. Multilevel CDA failure, where a staged anterior–posterior fusion may provide better long-term stability.
5. In cases with poor bone quality (e.g., osteoporosis), anterior plating alone is insufficient for stability.

### 4.4. Surgical Strategy Based on the Number of Levels Involved

The number of levels involved in cervical disc arthroplasty (CDA) failure is critical in determining the appropriate revision strategy. Single-level CDA failures can often be managed with an isolated anterior revision, whereas multilevel CDA failures require a more comprehensive approach, frequently involving posterior stabilization. The selection of anterior, posterior, or combined revision approaches depends on the extent of disease progression, biomechanical stability, and adjacent segment involvement (Table 1).

Single-level CDA failure typically occurs due to implant-related complications, including prosthesis subsidence, migration, or heterotopic ossification. These failures are generally localized to a single motion segment, preserving the integrity of adjacent levels. The impact on overall sagittal balance is minimal, so surgical revision strategies are relatively straightforward compared to multilevel failures. However, single-level failure may lead to progressive instability or adjacent segment degeneration if left unaddressed.

Two-level CDA failure represents a more advanced stage of degeneration than single-level cases. It is frequently associated with progressive kyphosis, adjacent segment disease (ASD), or multi-segment disc collapse. Due to the involvement of multiple motion segments, biomechanical instability becomes a significant concern. The increased load distribution over a broader cervical segment predisposes these patients to higher rates of subsidence, prosthesis failure, and pseudarthrosis.

Three-level CDA failure presents a complex surgical challenge due to a markedly increased risk of pseudarthrosis, significant kyphosis, and extensive multi-segment degeneration. Losing multiple motion segments results in severe instability, necessitating a more aggressive revision strategy. Anterior-only revision approaches are often inadequate due to the high mechanical stress placed on three-level fusion constructs, leading to an increased risk of hardware failure and nonunion.

## 5. Reoperation Strategy for CDA Failure at the Index Level

The reoperation strategy for CDA failure depends on whether the failure is localized to the index level, involves adjacent segments, or both. The following sections discuss revision approaches for both scenarios (Table 2) [22,23,24,25,26].

### 5.1. Conversion to ACDF or ACCF

**Figure 1:**
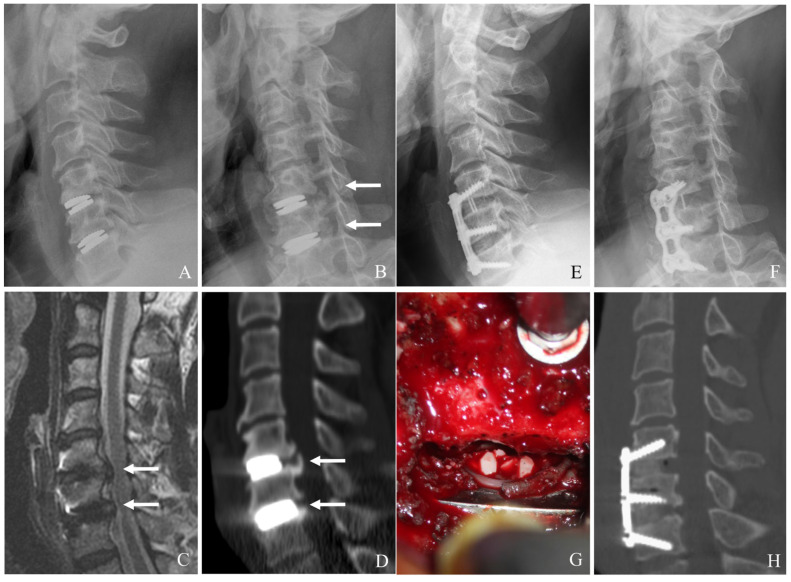
Plain X-rays (**A**,**B**), magnetic resonance image (**C**), and computed tomography (**D**) showing cervical disc arthroplasty at C5–6 and C6–7 with severe foraminal stenosis and posterior osteophytes (white arrows). The patient underwent removal of C5–6 and C6–7 prosthesis and revisional anterior cervical discectomy and fusion (**E**–**H**).

Conversion to ACDF is the most common approach when index-level failure is associated with severe heterotopic ossification (HO), significant subsidence, or mechanical instability [22,23,24,25,26]. This method involves an anterior approach to remove the failed prosthesis, debridement of scar tissues and HO, and neural decompression such as posterior longitudinal ligament (PLL) resection and uncoformaminotomy. An interbody fusion is then performed using appropriate graft material, augmented by anterior plating to achieve stability. ACDF provides robust stabilization and high fusion rates (often >90%). It can reliably alleviate symptoms (Figure 1). However, it sacrifices motion at the index level, potentially predisposing patients to further ASD.

#### 5.1.1. Indications for Conversion to ACDF or ACCF

Anterior reoperation is indicated in cases where CDA failure leads to symptomatic or structural issues that can be effectively managed from an anterior approach. Specific indications include the following:

(a) Device subsidence or migration (when the CDA implant subsides into the vertebral body or migrates anteriorly/posteriorly, leading to segmental instability or neural compression).
(b) HO (extensive HO that compromises the motion-preserving function of the prosthesis or causes direct compression of neural elements).
(c) Prosthesis loosening or malposition (mechanical prosthesis loosening due to poor initial fixation or bone quality, resulting in pain or neurological deficits).
(d) Persistent or recurrent radiculopathy/myelopathy (symptoms that persist despite initial CDA, often due to incomplete decompression, progressive degenerative changes, or foraminal stenosis).
(e) Infection or inflammatory reaction (localized infection at the prosthesis site or inflammatory responses, such as metallosis, necessitating implant removal, and debridement).
(f) Segmental instability without posterior involvement (instability confined to the index level that does not require additional posterior stabilization).
(g) Prosthesis fracture or mechanical failure (structural failure of the artificial disc components leading to mechanical dysfunction and pain).

#### 5.1.2. Revision Strategy of ACDF or ACCF

**Figure 2:**
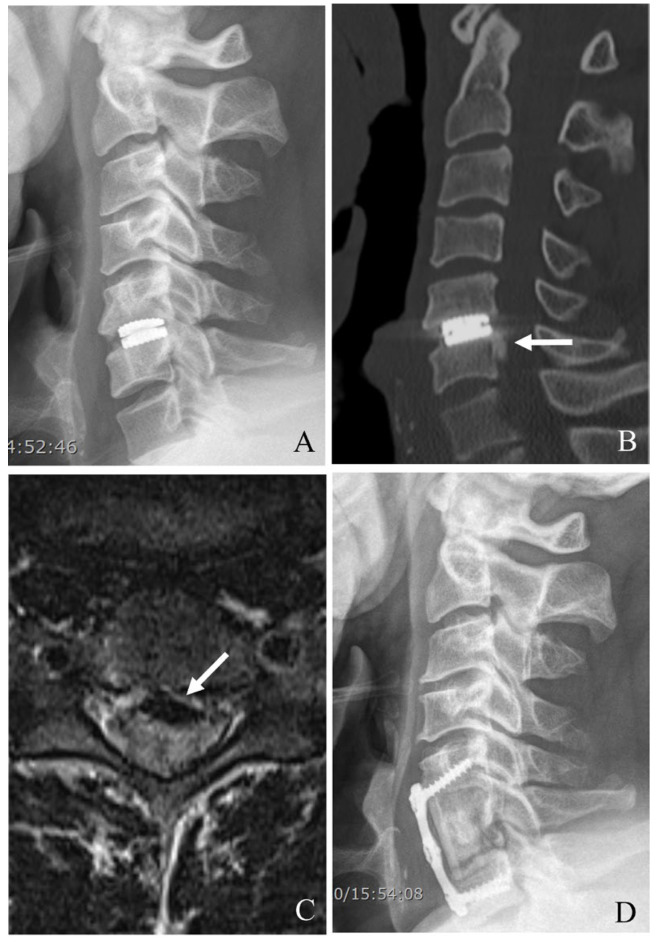
Plain X-rays (**A**), computed tomography (**B**), and magnetic resonance image (**C**) showing cervical disc arthroplasty at C5–6 and ossification of the posterior longitudinal ligament (OPLL) of C6 body (white arrow) causing cord compression. The patient underwent removal of the C5–6 prosthesis and OPLL and anterior cervical corpectomy and fusion (**D**).

For ACDF, after device removal, the intervertebral space is prepared for fusion. A structural graft (autograft, allograft, or synthetic cage) is placed, followed by anterior plating to provide immediate stability. In cases of severe bone loss or multilevel involvement, a corpectomy may be required, with the placement of a cage or graft to restore anterior column support for ACCF (Figure 2). The decision between two-level ACCF and three-level ACDF must be individualized based on the patient’s pathology, bone quality, and sagittal alignment needs. While corpectomy provides better deformity correction, it carries higher surgical risk and requires more extensive stabilization. In contrast, three-level ACDF is less invasive but has higher rates of pseudarthrosis, potentially leading to revision surgery. Understanding these nuances allows for more tailored surgical planning and improved patient outcomes [31,32].

### 5.2. Implantation Revision or Exchange (Redo CDA)

In select cases of CDA failure at the index level, a redo CDA can be considered as an alternative to ACDF [27,28,29]. This motion-preserving strategy is applicable when the patient’s anatomy and pathology permit the re-implantation of a new prosthesis after removing the failed device. Redo CDA is technically challenging and requires meticulous surgical planning. However, it offers the benefit of maintaining segmental motion, potentially reducing the risk of adjacent segment degeneration.

#### 5.2.1. Indications for Redo CDA

Redo CDA is appropriate only in carefully selected cases where motion preservation remains viable. Key indications include the following:

(a) Prosthesis malposition or mechanical failure without significant bone loss (failure due to malposition, mechanical dysfunction, or design flaws of the initial implant, where sufficient bone stock remains for secure re-implantation).
(b) Localized HO without severe degeneration (cases of heterotopic ossification (HO) causing mechanical dysfunction or neural compression with preserved disc space height and minimal facet joint arthropathy).
(c) Prosthesis subsidence without instability (mild-to-moderate subsidence of the prosthesis without evidence of gross instability or kyphotic deformity, where correction can be achieved with a new implant).
(d) Persistent radiculopathy/myelopathy due to incomplete decompression (when symptoms persist due to incomplete decompression at the index level with surrounding spinal segments and facet joints remaining intact).
(e) Young patients with high functional demands (in young, active patients where maintaining cervical motion is prioritized with spinal stability uncompromised).
(f) No evidence of infection, instability, or severe kyphosis (absence of infection, advanced spondylosis, or structural deformities that would otherwise necessitate fusion).

#### 5.2.2. Contraindications for Redo CDA

Redo CDA is not recommended in the following situations:

(a) Severe vertebral body erosion or bone loss (those with significant bone loss or poor bone quality that precludes secure anchoring of a new prosthesis).
(b) Segmental instability or kyphotic deformity (those with cervical instability or kyphosis requiring stabilization through fusion).
(c) Advanced degenerative changes (those with severe facet arthropathy, disc space collapse, or multilevel spondylotic changes that would compromise motion preservation).
(d) Infection or inflammatory conditions (those with active infection (e.g., discitis, osteomyelitis) or inflammatory conditions that would contraindicate implant placement_).
(e) Severe HO with ankylosis (those with extensive HO leading to auto-fusion of the segment that would make motion preservation impossible).

#### 5.2.3. Revision Strategy for Redo CDA

Redo CDA requires a meticulous approach to safely remove the failed implant and prepare the site for re-implantation. Although reusing the previous incision can minimize additional scarring, it poses challenges due to tissue fibrosis. Careful dissection around the prosthesis is required to avoid injury to vertebral bodies and neural structures. Specialized extraction tools may be necessary, especially in bony integration or heterotopic ossification cases. Any heterotopic bone is carefully excised, and the intervertebral space is cleaned to restore disc height and prepare endplates for the new prosthesis. Care must be taken to avoid excessive endplate removal, which could lead to further subsidence. A new artificial disc is selected based on intraoperative measurements. The device is implanted following standard arthroplasty techniques, ensuring proper alignment and fit.

### 5.3. Posterior Cervical Decompression and Fusion (PCDF)

**Figure 3:**
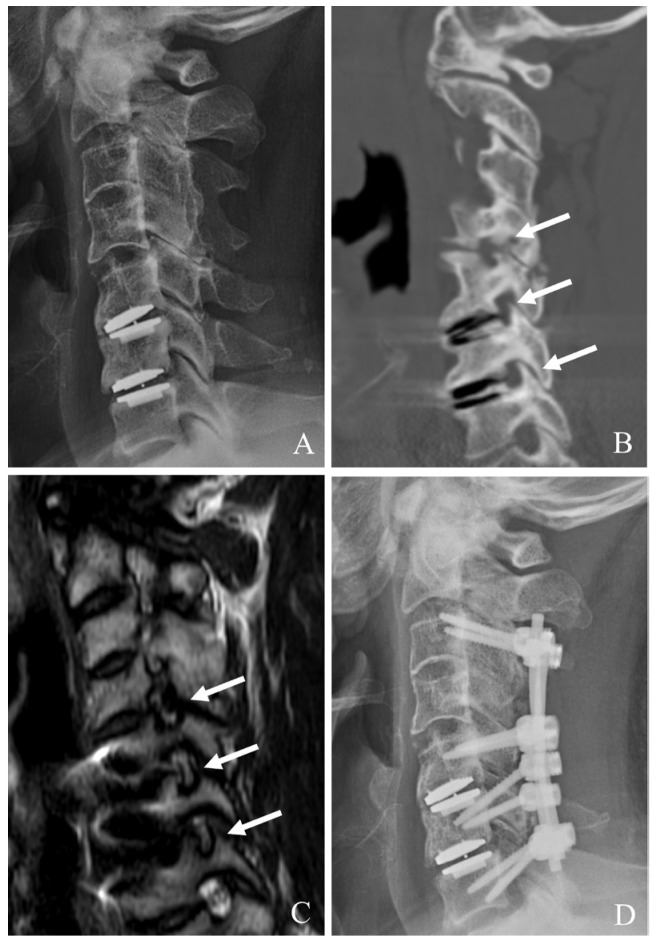
Plain X-rays (**A**), computed tomography (**B**), and magnetic resonance image (**C**) showing cervical disc arthroplasty at C5–6 and C6–7 and severe foraminal stenosis and posterior osteophytes (white arrows). The patient underwent C4–7 posterior cervical laminectomy, foraminotomy, and fusion with C3–7 pedicle screw fixation (**D**).

When CDA fails at the index level, PCDF is a well-established surgical option, particularly in cases where anterior reoperation is either contraindicated or insufficient [22,23,24,25,26]. PCDF provides robust neural decompression and stabilization, making it especially useful for cases with instability, deformity, or posterior element involvement (Figure 3). Although this approach is more invasive than anterior reoperations, it offers comprehensive management of those with complex failure mechanisms.

#### 5.3.1. Indications for PCDF

PCDF is typically indicated in the following scenarios:

(a) Segmental instability or kyphotic deformity (those with CDA failure leading to mechanical instability, dynamic spondylolisthesis, or progressive kyphosis, requiring posterior stabilization).
(b) Persistent or recurrent myelopathy with posterior compression (those with ongoing spinal cord compression due to posterior elements (e.g., ligamentum flavum hypertrophy, facet joint overgrowth) that is not adequately addressed by anterior approaches).
(c) Multilevel stenosis (when multilevel decompression is needed, particularly in patients with tandem stenosis (both anterior and posterior) or when adjacent levels exhibit significant pathology).
(d) HO with combined compression (those with severe heterotopic ossification (HO) leading to circumferential (anterior and posterior) compression that necessitates posterior decompression in addition to, or instead of, anterior revision).
(e) Failed anterior reoperation (e.g., pseudarthrosis after ACDF conversion) (patients who previously underwent anterior revision with ACDF but developed pseudarthrosis, resulting in instability and persistent symptoms).
(f) Contraindications to the anterior approach (cases where an anterior approach is contraindicated due to scar tissue from prior surgeries, anatomical considerations, or patient comorbidities such as esophageal or airway complications).
(g) Infectious or inflammatory conditions (when CDA failure is complicated by infection, posterior stabilization may be required after debridement to restore structural integrity).

#### 5.3.2. Contraindications for PCDF

While PCDF is a versatile approach, it may not be suitable for the following cases:

(a) Isolated anterior compression without instability (when the failure is solely due to anterior pathology (e.g., prosthesis subsidence, anterior migration), posterior decompression alone may be insufficient).
(b) Patients with severe medical comorbidities (those with increased surgical time and invasiveness of posterior procedures may not be appropriate for patients with significant medical risks).
(c) Mild symptoms with preserved stability (for cases with mild, non-progressive symptoms where conservative management or less invasive procedures (e.g., posterior foraminotomy) may suffice).

#### 5.3.3. Revision Strategy of PCDF

The lamina is removed at the index and adjacent levels to decompress the spinal cord, particularly in cases with central canal stenosis. If radiculopathy is present, nerve roots should be decompressed by widening of the neural foramen. For instrumentation and fusion, lateral mass or pedicle screw fixation is placed to provide rigid stabilization. The choice between lateral mass and pedicle screws depends on the surgeon’s preference and anatomical considerations. Autograft, allograft, or bone substitutes promote fusion across posterior elements. In cases with kyphotic deformity, instrumentation can be used to restore cervical lordosis intraoperatively.

### 5.4. Combined Anterior–Posterior Reoperation

In certain complex cases of CDA failure at the index level, a combined anterior–posterior reoperation may be necessary to achieve optimal decompression, stabilization, and symptom relief [22,23,24,25,26]. This approach is generally reserved for cases where a single anterior or posterior procedure alone cannot address the underlying pathology.

#### 5.4.1. Indications for Combined Anterior–Posterior Reoperation

**Figure 4:**
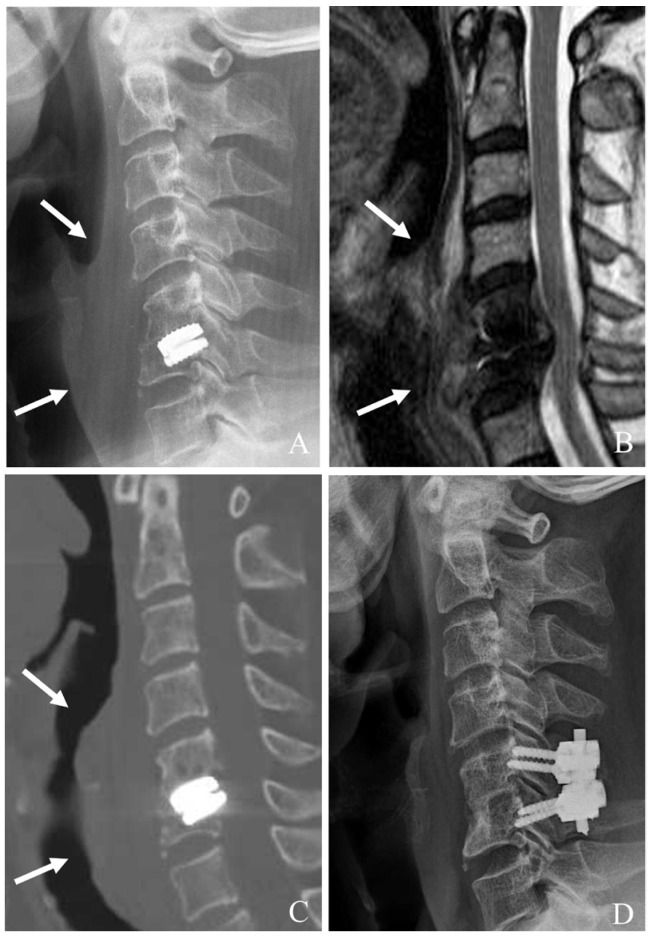
Plain X-rays (**A**), magnetic resonance image (**B**), and computed tomography (**C**) showing subsidence and retropulsion of cervical disc arthroplasty at C5–6 with retropharyngeal (white arrows) and epidural abscess causing cord compression. The patient underwent the removal of the C5–6 prosthesis and combined C5–6 anterior cervical discectomy and fusion and posterior fusion with lateral mass screws (**D**).

A combined approach is typically indicated in the following situations:

(a) Device-related failure with segmental instability (those with subsidence, migration, or loosening of the CDA device causing mechanical instability that cannot be adequately stabilized with either anterior or posterior fixation alone).
(b) Severe HO with multidirectional compression (those with extensive HO leading to both anterior and posterior compression of neural structures, necessitating decompression from both approaches).
(c) Cervical kyphotic deformity with instability (those with failure of CDA contributing to a kyphotic deformity that requires anterior correction (discectomy/corpectomy and fusion) combined with posterior stabilization to maintain alignment).
(d) Recurrent or persistent radiculopathy/myelopathy after prior surgery (when symptoms persist due to incomplete decompression from a prior anterior or posterior surgery, a comprehensive approach is needed to address both foraminal and central canal stenosis).
(e) Infectious or inflammatory complications (for cases with the infection involving the CDA device, complete debridement and stabilization from both anterior and posterior approaches might be necessary to eradicate the infection and restore spinal integrity) (Figure 4).

#### 5.4.2. Revision Strategy of Combined Anterior–Posterior Reoperation

The procedure can be performed as a single-stage surgery or staged over separate operations, depending on patient factors, the complexity of the case, and intraoperative findings.

(a) Step 1, anterior approach: The first step involves removing the failed CDA device. This may be technically challenging due to bone overgrowth or device integration with the vertebral bodies. Depending on the degree of degeneration or bone loss, either a discectomy or corpectomy is performed to decompress the spinal cord and nerve roots. A structural graft or cage (e.g., PEEK, titanium, or autograft) is placed in the intervertebral space, followed by anterior plating to restore disc height and maintain alignment.
(b) Step 2, posterior approach: A laminectomy may be performed for additional decompression of the spinal cord for cases with residual posterior compression. In motion-preserving cases, laminoplasty may be considered if stability is adequate. For posterior instrumentation, lateral mass or pedicle screw fixation is used to provide additional stabilization, particularly in cases with significant preoperative instability or deformity. Bone grafting is applied for posterior fusion to achieve a solid fusion across posterior elements, ensuring long-term stability.

## 6. Reoperation Strategy for CDA Failure at Adjacent Level (ASD After CDA)

ASD is a well-documented complication following both CDA and ACDF. Although CDA is designed to preserve motion and theoretically reduce the incidence of ASD compared to fusion, degenerative changes can still occur at levels adjacent to the index CDA, necessitating reoperation. Surgical management of ASD after CDA can involve anterior, posterior, or combined anterior–posterior approaches, depending on the pathology, severity, and anatomical considerations [13,14,15,16,17,18].

When ASD develops after a prior CDA, anterior reoperation is often the first-line approach due to its direct access to the site of pathology. Anterior reoperation can be performed as ACDF or a Redo CDA at the adjacent level, depending on the patient’s specific conditions, anatomy, and treatment goals.

Posterior reoperation offers several surgical options depending on the severity, location, and type of pathology. Posterior approaches are beneficial for addressing posterior element degeneration, multilevel stenosis, or cases where anterior approaches are contraindicated. These posterior reoperation strategies include foraminotomy or decompression alone, PCDF, and laminoplasty.

### 6.1. ACDF for ASD After CDA

ACDF is the most commonly performed anterior reoperation for ASD following CDA, especially when motion preservation is no longer feasible [22,23,24,25,26]. The index-level CDA can be preserved if it remains functional and stable. If the index-level prosthesis shows signs of failure, it may be removed, and the level converted to fusion, creating a hybrid construct. Key indications include the following:

(a) Severe degeneration at adjacent levels, such as advanced disc degeneration, osteophyte formation, or facet joint arthropathy, precludes the successful placement of a new prosthesis.
(b) Segmental instability or kyphosis, such as hyper-mobility, spondylolisthesis, and progressive kyphotic deformity at adjacent levels, that requires stabilization.
(c) Recurrent or persistent myelopathy/radiculopathy due to anterior compression from large disc herniations, osteophytes, or OPLL that necessitates fusion for definitive decompression.
(d) Previous CDA failure or motion-preservation contraindications (when prior CDA at the index level fails due to heterotopic ossification, subsidence, or prosthesis migration, fusion is a safer option at the adjacent level).
(e) Poor bone quality or osteoporosis (in patients with compromised bone quality, fusion offers more reliable stabilization than motion-preserving techniques).
(f) Multilevel disease (for cases where multiple adjacent levels are affected, ACDF may be more practical and effective).

### 6.2. Redo CDA for ASD After CDA

**Figure 5:**
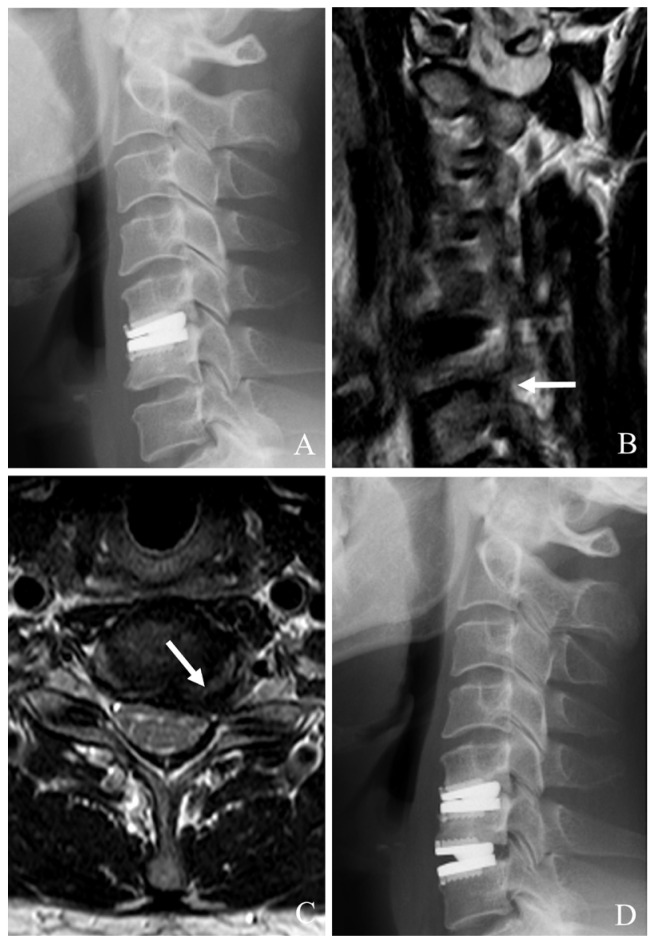
Plain X-rays (**A**), magnetic resonance image (**B**,**C**) showing kyphosis of C5–6 prosthesis and C6–7 adjacent segment disease with left foraminal stenosis and disc herniation (white arrows). The patient underwent removal of C6–7 redo cervical disc arthroplasty (**D**).

Redoing CDA at the adjacent level is a motion-preserving option for carefully selected patients. It aims to maintain cervical mobility and reduce the long-term risk of adjacent segment degeneration (Figure 5) [27,28,29].

If the original CDA functions well, the original prosthesis is left in place. The original CDA can be converted to fusion if necessary while maintaining motion at the newly operated adjacent level. Key indications include the following:

(a) Mild-to-moderate degeneration at adjacent levels (those with disc degeneration at adjacent levels without severe osteophyte formation or facet arthropathy).
(b) No significant instability or deformity (those without segmental kyphosis, instability, or dynamic spondylolisthesis at adjacent levels).
(c) Preserved bone quality (those with sufficient bone stock and vertebral endplate integrity to support a new prosthesis).
(d) Young patients with high functional demands (young and active individuals who prioritize motion preservation for occupational or lifestyle reasons).
(e) Successful functioning of index-level CDA (when the initial CDA remains functional without evidence of heterotopic ossification or prosthesis failure at the index level).
(f) No significant posterior pathology (redoing CDA is not ideal if posterior elements, such as facet joints or ligamentum flavum, contribute significantly to neural compression).

### 6.3. Posterior Foraminotomy or Decompression Alone for ASD After CDA

Foraminotomy or decompression without fusion is a motion-preserving posterior procedure designed to relieve nerve root compression while maintaining spinal mobility. This approach is ideal for patients with focal radiculopathy due to foraminal stenosis without instability or significant deformity [22,23,24,25,26]. Keyhole foraminotomy removes part of the lamina and medial portion of the facet joint to enlarge the neural foramen and decompress the affected nerve root. Care is taken to preserve at least 50% of the facet joint to maintain spinal stability. If central or lateral recess stenosis is present, limited laminectomy or laminotomy can be performed without fusion to decompress the spinal cord. Key indications include the following:

(a) Unilateral or bilateral foraminal stenosis at adjacent levels (those with compression of nerve roots due to foraminal narrowing from facet hypertrophy, osteophyte formation, or disc bulging at levels adjacent to the CDA).
(b) Isolated radiculopathy without myelopathy (patients presenting with radicular symptoms (e.g., arm pain, numbness, or weakness) without signs of spinal cord compression).
(c) Preserved segmental stability (those without cervical instability, kyphosis, or dynamic spondylolisthesis on flexion–extension radiographs).
(d) Good motion preservation at the index CDA level (those whose initial CDA is functioning well without signs of failure or adjacent instability).
(e) Patients preferring motion preservation (Young, active patients who wish to avoid fusion and maintain spinal mobility).

### 6.4. Posterior Cervical Decompression and Fusion (PCDF) for ASD After CDA

PCDF is a comprehensive surgical approach designed to address both neural compression and spinal instability. It is indicated that ASD leads to significant degenerative changes, instability, or deformity at adjacent levels [22,23,24,25,26]. Laminectomy is necessary to remove the laminae to decompress the spinal cord in cases of central canal stenosis. Foraminotomy or decompression of nerve roots needs to be performed if radiculopathy is present. For instrumentation and fusion, lateral mass or pedicle screws are inserted into the lateral masses or pedicles for robust stabilization. Autograft, allograft, or bone substitutes are applied to achieve fusion across the posterior elements. Instrumentation is adjusted to restore cervical lordosis and correct kyphotic deformities. Indications of PCDF include the following:

(a) Cervical instability at adjacent levels (those with dynamic spondylolisthesis, hypermobility, or segmental kyphosis identified on flexion–extension radiographs).
(b) Multilevel cervical stenosis (those with compression of the spinal cord or nerve roots across multiple adjacent levels that would require extensive decompression and stabilization).
(c) Persistent or progressive myelopathy (those with symptoms of spinal cord dysfunction (e.g., gait disturbance, hand clumsiness) due to central canal stenosis at adjacent segments).
(d) Failed prior anterior surgery (when anterior approaches are contraindicated due to scar tissue, infection, or failure of previous anterior surgeries).
(e) Severe degenerative changes in facet joints or posterior elements (those with advanced facet arthropathy, ligamentum flavum hypertrophy, or OPLL).
(f) CDA Failure at the index level with adjacent segment Involvement (when both the index CDA and adjacent levels show signs of failure or instability that would necessitate posterior stabilization).

### 6.5. Laminoplasty for ASD After CDA

**Figure 6:**
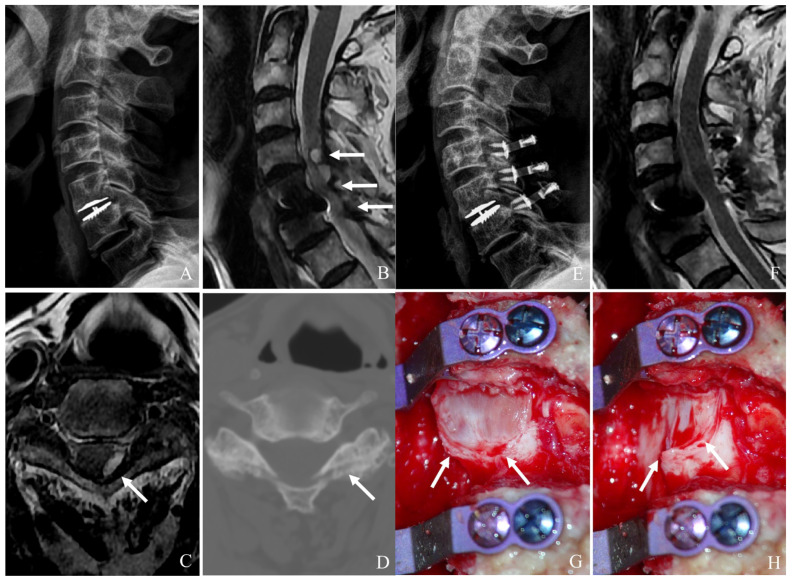
Plain X-rays (**A**), magnetic resonance image (**B**,**C**), computed tomography (**D**), and clinical photo (**G**) showing hypertrophy of ligamentum flavum at C4–6 and facet cyst at C4–5 causing cord compression (white arrows). The patient underwent C4accept–6 open-door laminoplasty and removal of facet cyst, leading to complete cord decompression (**E**,**F**,**H**).

Laminoplasty is a motion-preserving posterior decompression technique particularly useful for multilevel cervical stenosis without significant instability. It expands the spinal canal while maintaining the integrity of posterior elements (Figure 6A–H) [22,23,24,25,26]. Key indications include the following:

(a) Multilevel cervical stenosis without instability (those with central canal stenosis across multiple adjacent levels without segmental kyphosis or instability).
(b) Myelopathy with preserved sagittal alignment (those with cervical myelopathy and normal or mildly lordotic alignment).
(c) Patients prioritizing motion preservation (young, active patients seeking to maintain cervical mobility while addressing neural compression).
(d) Mild posterior element degeneration (those with minimal facet arthropathy and ligamentum flavum hypertrophy.
(e) Contraindications to anterior surgery (those with high surgical risk for anterior approaches due to prior surgeries, scar tissue, or medical comorbidities.

### 6.6. Combined Anterior–Posterior Reoperation for ASD After CDA

A combined anterior–posterior approach is reserved for complex cases where neither approach alone can adequately address the pathology [22,23,24,25,26]. Key indications include the following:

(a) Severe multilevel degeneration with instability (degenerative changes at multiple adjacent levels with significant instability, requiring both anterior decompression and posterior stabilization).
(b) Kyphotic deformity involving adjacent levels (severe kyphosis extending into adjacent segments that necessitate anterior column support and posterior tension band restoration).
(c) Failed prior surgeries with persistent symptoms (those with previous anterior or posterior surgeries that fail to relieve symptoms or result in further instability).
(d) Circumferential compression (anterior and posterior) (cases involving both anterior disc herniation/osteophytes and posterior ligamentum hypertrophy or facet degeneration).

For staging of surgery, the combined approach may be performed as a single-stage procedure or staged over separate operations, depending on the patient’s conditions and the complexity of the surgery. The anterior surgery is typically the first step and involves procedures such as discectomy or corpectomy to remove compressive anterior pathology at adjacent levels. Depending on the pathology, either fusion (ACDF) or motion-preserving CDA is performed at adjacent levels. To correct sagittal alignment, lordosis restoration must be performed through anterior column reconstruction.

As part of the posterior surgery, laminectomy/foraminotomy is performed for additional decompression of the spinal cord and nerve roots as needed. Posterior fusion and instrumentation with lateral mass or pedicle screws are necessary to stabilize the spine, particularly in cases with instability or deformity correction.

## 7. Long-Term Patient-Reported Outcomes Post-Revision

Evaluating long-term outcomes following cervical disc arthroplasty (CDA) revision surgery is critical for understanding different surgical approaches’ effectiveness, durability, and patient satisfaction. Patient-reported outcome measures (PROMs) provide valuable insights into pain levels, functional recovery, and overall quality of life following revision procedures. This Section reviews key PROMs, including the Neck Disability Index (NDI), Visual Analog Scale (VAS), and the Short Form-36 (SF-36), and compares reported outcomes based on different revision strategies.

Key findings of long-term PRO following revision surgery include (Table 3): (1) Conversion to ACDF results in significant functional improvement (NDI) and pain relief (VAS decrease of 3–5 points), making it a highly effective revision strategy. (2) Redo CDA preserves motion but has less predictable outcomes, with some patients experiencing persistent symptoms due to residual degeneration or implant-related factors. (3) Posterior Decompression and Fusion (PCDF) provides robust pain relief and is particularly effective in cases with posterior compression or multilevel stenosis, but it may lead to postoperative stiffness. (4) Combined Anterior–Posterior Surgery offers the most significant improvement in function and pain relief, but the higher surgical morbidity and more extended recovery period should be carefully considered [22,23,24,25,26,27,28,29].

## 8. Conclusions

Revision surgery for CDA remains challenging due to the lack of high-level evidence and standardized guidelines. While conversion to ACDF is the most commonly performed revision, alternative strategies such as redo CDA, PCDF, and combined anterior–posterior approaches may be appropriate in select cases based on patient-specific factors. Currently, surgical decision-making relies heavily on surgeon experience and retrospective data, underscoring the need for prospective studies and randomized controlled trials (RCTs) to guide best practices. Additionally, long-term PROMs remain underreported, limiting our understanding of the durability and efficacy of different revision strategies. Future research should focus on standardizing revision protocols through multicenter studies, integrating both clinical and biomechanical perspectives to optimize patient outcomes. Until more robust data are available, individualized, evidence-based surgical decision-making remains essential in managing CDA failure.

## Figures and Tables

**Table 1:** Comparison of Revision Strategies Based on Number of Levels Involved.

Number of Failed Levels	Common Failure Mechanism	Preferred Revision Strategy
Single-Level CDA Failure	Implant migration, Subsidence, Localized heterotopic ossification	Anterior ACDF, Posterior fusion (select cases)
Two-Level CDA Failure	Adjacent segment disease, Progressive kyphosis	Anterior two-level ACDF, Anterior–posterior fusion (if instability is present)
Three-Level CDA Failure	Severe kyphosis, Multilevel adjacent segment disease	Three-level ACDF (limited cases), Combined anterior–posterior fusion for Optimal stability

**Table 2:** Indications for Different CDA Revision Strategies.

Revision Strategy	Indications
Conversion to ACDF	Device subsidence or migration leading to segmental instability or neural compression
	Extensive heterotopic ossification (HO) compromises motion preservation or causes neural compression
	Prosthesis loosening or malposition causing mechanical dysfunction Persistent or recurrent radiculopathy/myelopathy due to incomplete decompression of Foraminal stenosis Infection or inflammatory reaction necessitating implant removal and debridement Segmental instability without posterior involvement Prosthesis fracture or mechanical failure
Redo CDA	Prosthesis malposition or mechanical failure without significant bone loss Localized HO without severe degeneration, preserving disc space height Prosthesis subsidence without instability or kyphotic deformity
	Persistent radiculopathy/myelopathy due to incomplete decompression with intact surrounding segments Young patients with high functional demands where motion preservation is prioritized No evidence of infection, instability, or severe kyphosis
Posterior Decompression and Fusion (PCDF)	Segmental instability or kyphotic deformity requiring posterior stabilization Persistent or recurrent myelopathy due to posterior compression (ligamentum flavum hypertrophy, facet joint overgrowth)
	Multilevel stenosis requiring decompression beyond the index level Severe HO leading to circumferential (anterior and posterior) neural compression Failed anterior reoperation (e.g., pseudarthrosis after ACDF conversion) Contraindications to anterior approach due to previous scarring, esophageal complications, or comorbidities
	Infection requiring stabilization after debridement
CombinedAnterior–Posterior Surgery	Device-related failure with segmental instability that cannot be addressed with anterior or posterior fixation alone Severe HO with multidirectional compression requiring decompression from both approaches. Cervical kyphotic deformity requiring anterior correction and posterior stabilizationRecurrent or persistent radiculopathy/myelopathy after previous surgery due to incomplete decompression Infection necessitating complete debridement and dual stabilization

**Table 3:** Comparison of Long-Term Patient-Reported Outcomes by Revision Type.

Revision Type	NDI Improvement	VAS Pain Reduction	SF-36 Score Change
Conversion to ACDF	Significant improvement in functional scores (>30% reduction in NDI)	Marked pain reduction (VAS decrease of 3–5 Points)	Moderate improvement in physical and mental health scores
Redo CDA	Variable improvement, depends on preoperative pathology	Mild-to-moderate pain reduction, but the risk of residual symptoms	Better preservation of motion, but long-term benefits unclear
Posterior Decompression and Fusion (PCDF)	Greater NDI reduction in cases with severe myelopathy	Significant pain relief, particularly in radiculopathy cases	Lower SF-35 scores initially due to postoperative stiffness, but stable long-term
Combined Anterior–Posterior Surgery	Largest NDI improvement due to the correction of deformity and instability	Principal VAS pain reduction, though higher perioperative morbidity	More sustained SF-35 gains due to comprehensive stabilization

## Data Availability

Not applicable.

## Abbreviations

The following abbreviations are used in this manuscript:

ACCF	Anterior Cervical Corpectomy and Fusion
ACDF	Anterior Cervical Discectomy and Fusion
ASD	Adjacent Segment Disease
CDA	Cervical Disc Arthroplasty
PCDF	Posterior Cervical Decompression and Fusion

## References

[1] Steinberger J., Qureshi S. (2020). Cervical disc replacement. Neurosurg. Clin. N. Am..

[2] Peng Z., Hong Y., Meng Y., Liu H. (2022). A meta-analysis comparing the short- and mid- to long-term outcomes of artificial cervical disc replacement(ACDR) with anterior cervical discectomy and fusion (ACDF) for the treatment of cervical degenerative disc disease. Int. Orthop..

[3] Wahood W., Yolcu Y.U., Kerezoudis P., Goyal A., Alvi M.A., Freedman B.A., Bydon M. (2020). Artificial discs in cervical disc replacement: A meta-analysis for comparison of long-term outcomes. World Neurosurg..

[4] Nunley P.D., Coric D., Frank K.A., Stone M.B. (2018). Cervical disc arthroplasty: Current evidence and real-world application. Neurosurgery.

[5] Makhni M.C., Osorio J.A., Park P.J., Lombardi J.M., Riew K.D. (2019). Cervical disc arthroplasty: Tips and tricks. Int. Orthop..

[6] Qi M., Xu C., Liu Y., Cao P., Wang X., Chen H., Yuan W. (2023). Comparison of clinical outcomes between cervical disc arthroplasty and anterior cervical discectomy and fusion for the treatment of single-level cervical spondylosis: A 10-year follow-up study. Spine J..

[7] Kim K., Hoffman G., Bae H., Redmond A., Hisey M., Nunley P., Jackson R., Tahernia D., Araghi A. (2021). Ten-year outcomes of 1- and 2-level cervical disc arthroplasty from the Mobi-C investigational device exemption clinical trial. Neurosurgery.

[8] Hui N., Phan K., Cheng H.M.K., Lin Y.-H., Mobbs R.J. (2020). Complications of cervical total disc replacement and their associations with heterotopic ossification: A systematic review and meta-analysis. Eur. Spine J..

[9] Joaquim A.F., Lee N.J., Lehman R.A., Tumialán L.M., Riew K.D. (2020). Osteolysis after cervical disc arthroplasty. Eur. Spine J..

[10] Scott-Young M., Rathbone E., Grierson L. (2022). Midterm osteolysis-induced aseptic failure of the M6-C™ cervical total disc replacement secondary to polyethylene wear debris. Eur. Spine J..

[11] Price R.L., Coric D., Ray W.Z. (2021). Cervical total disc replacement. Complications and complication avoidance. Neurosurg. Clin. N. Am..

[12] Kong L., Ma Q., Meng F., Cao J., Yu K., Shen Y. (2017). The prevalence of heterotopic ossification among patients after cervical artificial disc replacement A systematic review and meta-analysis. Medicine.

[13] Parish J.M., Asher A.M., Coric D. (2021). Adjacent-segment disease following spinal arthroplasty. Neurosurg. Clin. N. Am..

[14] Dong L., Xu Z., Chen X., Wang D., Li D., Liu T., Hao D. (2017). The change of adjacent segment after cervical disc arthroplasty compared with anterior cervical discectomy and fusion: A meta-analysis of randomized controlled trials. Spine J..

[15] Burkhardt B.W., Baumann L., Simgen A., Wagenpfeil G., Hendrix P., Reith W., Oertel J.M. (2022). Long-term follow-up MRI shows no hastening of adjacent segment degeneration following cervical disc arthroplasty. Sci. Rep..

[16] Liang Y., Qian Y., Xia W., Guo C., Zhu Z., Liu H., Xu S. (2024). Adjacent segment degeneration after single- and double-level cervical total disc replacement: A cohort with an over 12-year follow-up. Eur. Spine J..

[17] Wang X., Meng Y., Liu H., Guo C., Zhu Z., Liu H., Xu S. (2020). Association of cervical sagittal alignment with adjacent segment degeneration and heterotopic ossification following cervical disc replacement with Prestige-LP disc. J. Orthop. Surg..

[18] Xu S., Liang Y., Zhu Z., Qian Y., Liu H. (2018). Adjacent segment degeneration or disease after cervical total disc replacement: A meta-analysis of randomized controlled trials. J. Orthop. Surg. Res..

[19] Skeppholm M., Henriques T., Tullberg T. (2017). Higher reoperation rate following cervical disc replacement in a retrospective, long-term comparative study of 715 patients. Eur. Spine J..

[20] Zhong Z.M., Zhu S.Y., Zhuang J.S., Wu Q., Chen J.-T. (2016). Reoperation after cervical disc arthroplasty versus anterior cervical discectomy and fusion: A meta-analysis. Clin. Orthop. Relat. Res..

[21] Chang K.E., Pham M.H., Hsieh P.C. (2017). Adjacent segment disease requiring reoperation in cervical total disc arthroplasty: A literature review and update. J. Clin. Neurosci..

[22] Park J.B., Chang H., Yeom J.S., Suk K.-S., Lee D.-H., Lee J.C. (2016). Revision surgeries following artificial disc replacement of cervical spine. Acta Orthop. Traumatol. Turc..

[23] Reinkec O.A., Radkeb J., Fingera T., Bayerl S., Vajkoczy P., Meyer B. (2017). Revision surgery for cervical artificial disc: Surgical technique and clinical results. Clin. Neurol. Neurosurg..

[24] Blumenthal S.L., Ohnmeiss D.D., Courtois E.C., Guyer R.D., Zigler J.E., Shellock J.L. (2024). Treatment of failed cervical total disc replacements in a series of 53 cases and description of a management strategy. Eur. Spine J..

[25] Joaquim A.F., Lee N.J., Riew K.D. (2021). Revision surgeries at the index level after cervical disc arthroplasty—A systematic review. Neurospine.

[26] Blumenthal S.L., Griffin C., Courtois E.C., Guyer R.D., Zigler J.E., Shellock J.L., Ohnmeiss D.D. (2024). Removals and revisions of cervical total disc replacement devices in a consecutive series of 1626 patients beginning with the first case experience in 2003. Spine.

[27] Lee N.J., Joaquim A.F., Boddapati V., Mathew J., Park P., Kim J.S., Sardar Z.M., Lehman R.A., Riew K.D. (2022). Revision anterior cervical disc arthroplasty: A national analysis of the associated indications, procedures, and postoperative outcomes. Global Spine J..

[28] Bin S., Xiangwang H., Sheng X., Xiao S., Xiang T., Liu X., Zhang Y., Liu B. (2017). Artificial cervical disk replacement for the treatment of adjacent segment disease after anterior cervical decompression and fusion. Clin. Spine Surg..

[29] Rajakumar D.V., Hari A., Krishna M., Konar S., Sharma A. (2017). Adjacent-level arthroplasty following cervical fusion. Neurosurg. Focus..

[30] Kato S., Ganau M., Fehlings M.G. (2018). Surgical decision-making in degenerative cervical myelopathy—Anterior versus posterior approach. J. Clin. Neurosci..

[31] Lofrese G., Trungu S., Scerrati A., De Bonis P., Cultrera F., Mongardi L., Montemurro N., Piazza A., Miscusi M., Tosatto L. (2023). Two-level corpectomy and fusion vs. three-level anterior cervical discectomy and fusion without plating: Long-term clinical and radiological outcomes in a multicentric retrospective analysis. Life.

[32] Opara J., Odzimek M. (2024). Cervical spondylotic myelopathy-diagnostics and clinimetrics. Diagnostics.

